# Genetic background modulates phenotypic expressivity in OPA1 mutated mice, relevance to DOA pathogenesis

**DOI:** 10.3389/fnmol.2023.1241222

**Published:** 2023-09-06

**Authors:** Djamaa Atamena, Venu Gurram, Petnoï Petsophonsakul, Farnoosh Khosrobakhsh, Macarena S. Arrázola, Marlène Botella, Bernd Wissinger, Marion Szelechowski, Pascale Belenguer

**Affiliations:** ^1^Centre de Recherches sur la Cognition Animale (CRCA), Centre de Biologie Intégrative (CBI), Université Toulouse III, CNRS, Toulouse, France; ^2^Department of Biological Science, University of Kurdistan, Sanandaj, Iran; ^3^Institute for Ophthalmic Research, Centre for Ophthalmology, University of Tübingen, Tübingen, Germany

**Keywords:** optic atrophy, OPA1, mitochondria, genetic modifiers, disease severity, mouse strains

## Abstract

Dominant optic atrophy (DOA) is mainly caused by OPA1 mutations and is characterized by the degeneration of retinal ganglion cells (RGCs), whose axons form the optic nerve. The penetrance of DOA is incomplete and the disease is marked by highly variable expressivity, ranging from asymptomatic patients to some who are totally blind or who suffer from multisystemic effects. No clear genotype–phenotype correlation has been established to date. Taken together, these observations point toward the existence of modifying genetic and/or environmental factors that modulate disease severity. Here, we investigated the influence of genetic background on DOA expressivity by switching the previously described DOA mouse model bearing the c.1065 + 5G → A *Opa1* mutation from mixed C3H; C57BL/6 J to a pure C57BL/6 J background. We no longer observed retinal and optic nerve abnormalities; the findings indicated no degeneration, but rather a sex-dependent negative effect on RGC connectivity. This highlights the fact that RGC synaptic alteration might precede neuronal death, as has been proposed in other neurodegenerative diseases, providing new clinical considerations for early diagnosis as well as a new therapeutic window for DOA. Furthermore, our results demonstrate the importance of secondary genetic factors in the variability of DOA expressivity and offer a model for screening for aggravating environmental and genetic factors.

## 1. Introduction

Mitochondria are involved in numerous crucial functions, including energy metabolism, calcium homeostasis, and apoptosis, making them essential for cell homeostasis and survival. This is especially true in the case of neurons, which rely heavily on mitochondria to generate the energy and metabolites necessary to maintain their activity and plasticity. Moreover, mitochondria are highly dynamic organelles that fuse, divide, and move, thus being distributed across the different neuronal compartments in order to carry out their functions (Rangaraju et al., 2019). In turn, mitochondrial dysfunctions and impaired mitochondrial dynamics are associated with a wide range of diseases, including neurodegenerative diseases (Bertholet et al., 2016). In this context, mutations in the *OPA1* gene, which codes for a mitochondrial protein that controls mitochondrial fusion, have been linked to dominant optic atrophy (DOA) (Delettre et al., 2000).

Clinical manifestations of DOA include progressive bilateral and symmetrical vision loss starting from early childhood, as well as central scotoma, central or paracentral visual field defects, optic nerve pallor, and blue–yellow dyschromatopsia (Lenaers et al., 2021). The main targets of the disease are the retinal ganglion cells (RGCs), whose axons form the optic nerve. In addition, extra-ocular manifestations are described in 20% of patients suffering from syndromic forms (DOA^+^), including sensorineural deafness, ataxia, and peripheral neuropathy (Lenaers et al., 2021). DOA has a prevalence of 1:10000 to 1:50000, and its penetrance is incomplete. Most DOA patients (60–80%) harbor heterozygous mutations in *OPA1* and, to date, more than 300 pathogenic variants have been identified (Le Roux et al., 2019). No clear evidence for any genotype–phenotype correlation has been found, with the exception of missense mutations in the GTPase domain of OPA1, which have been proposed to be associated with DOA^+^ (Ham et al., 2019). In addition, the disease is characterized by marked variability of expressivity within and between families, suggesting the presence of genetic and/or environmental factors that exert strong influence, along with the primary mutation, on the manifestation of the disease. Thus far, no positive or negative genetic or environmental factors that may modulate DOA expressivity have been clearly identified.

Three mouse models have been developed to better understand DOA pathophysiology, and in particular, to assess the impact of *Opa1* mutations on the retina and optic nerves as well as on visual abilities (Alavi et al., 2007; Davies et al., 2007; Sarzi et al., 2012). As in the case of human patients, these DOA mouse models do not all display the same phenotypic features; instead, they exhibit manifestations ranging from mild and specific visual failure to a very severe syndromic form of the disease. Interestingly, although different mutations are present in the three mouse models, these mutations lead to the absence of OPA1 mutated protein in the mouse tissues, resulting in a state of haploinsufficiency that mimics the main pathogenic mechanism of DOA (Lenaers et al., 2021). Hence, the observed differences in phenotypes cannot be explained by the primary mutation. However, the three mouse models have been developed in different and/or mixed genetic backgrounds. Therefore, modifying secondary genetic factors could account for the different phenotypes. To explore the influence of genetic background on the clinical outcomes of OPA1-related diseases, we moved the mouse model (OPA1^329-355del^) developed by Alavi and colleagues (Alavi et al., 2007), which carries the c.1065 + 5G → A *Opa1* mutation, from a mixed genetic background (C3H; C57BL/6 J) to a pure genetic background (C57BL/6 J). We evaluated the impact of this genetic change on retinal and optic nerve integrity. Overall, our data provide evidence that (1) genetic background contributes to the phenotypic variability observed in DOA; (2) alteration of the connectivity of the RGCs is involved in DOA pathogenesis; and (3) our “new” model offers an exciting opportunity to analyze genetic and environmental contributions to DOA pathogenesis.

## 2. Materials and methods

### 2.1. Animals

Mice were bred and reared in the animal facility at the CBI (approval number 31-555-011) under a standard 12/12 h light–dark cycle and were provided with *ad libitum* water and food. All procedures were conducted in accordance with European Union policies (2010/63/EU). According to European regulations, only the use of animals in experimental procedures requires the approval of an ethics committee. Article R214-89 of the French Rural Code specifies that the killing of animals for the sole purpose of using their organs or tissues is not considered an experimental procedure.

Male mice carrying the *Opa1* c.1065 + 5G → A mutation from Alavi and collaborators (Alavi et al., 2007) were crossed over more than 10 generations with C57BL/6 J female mice (Janvier Labs) to maintain a homogenous genetic background (OPA1^329-355del^ C57BL/6 J mouse model). The genotype of the mice was systematically checked by PCR followed by restriction enzyme digestion analysis (as the mutation removes an AccI restriction site), after birth and after euthanasia of each mouse.

### 2.2. Western blot

Retinas were prepared from eyes freshly isolated from euthanized mice as previously described (Escher and Schorderet, 2011). These were incubated for 30 min (with vortex every 5 min) in lysis buffer containing 50 mM Tris–HCl pH 7.5, 250 mM NaCl, 5 mM ethylenediaminetetraacetic acid (EDTA), 5 mM ethylene glycol tetraacetic acid (EGTA), 1 mM dithiothreitol, 0.1% Triton X-100, 0.1% SDS, 1% deoxycholate, 1% NP-40, and protease inhibitors (“Complete” protease inhibitor cocktail, Roche Applied Science). Next, centrifugation was performed for 15 min at 15000 g, 4°C, and the supernatant was collected. Protein concentration was determined using the Bradford assay. Subsequently, 20 to 50 μg of protein was loaded onto a 4–15% polyacrylamide gel and transferred onto a nitrocellulose membrane. Membranes were incubated overnight at 4°C with anti-OPA1 (Bioscience 611,112, RRID AB 398423, 1/1000), anti-TOMM20 (Abcam 186,735, RRID AB 2889972, 1/10000), and anti-αTUBULIN (Sigma T5168, RRID AB 477579, 1/500) primary antibodies, followed by incubation with appropriate HRP-conjugated secondary antibodies (Abcam 6,721, RRID AB 477579 or 6,789, RRID AB 955439, 1/10000). After enhanced detection via chemiluminescence, the signal was quantified using the Image Lab software (Biorad, RRID SCR 014210).

### 2.3. Immunohistochemistry

Eyes were freshly isolated from euthanized mice as previously described (Escher and Schorderet, 2011). The cornea and lens were removed and the eyecups were fixed in 4% paraformaldehyde overnight, then cryopreserved overnight in PBS-30% sucrose at 4°C, embedded in optimal cutting temperature medium (Tissue-Tek 4,583) and stored at −20°C. Frozen 10 μm-thick sections (Cryostat Leica CM1950) were mounted on SuperFrost Plus slides (ThermoScientific, J1800AMNZ). After rinsing with PBS, retinal sections were permeabilized with 0.25% TritonX-100 in PBS for 20 min at room temperature (RT) and then incubated for 2 h in a blocking solution containing 3% bovine serum albumin, 5% goat serum, and 0.1% Triton X-100 in PBS at RT. Primary antibodies directed against Brn3-a (Millipore MAB1585, RRID AB94166, 1/200), GFAP (Abcam 7,260, RRID AB 305808, 1/10000), Iba1 (Wako 01919741, RRID AB 839504, 1/1000), and synaptophysin (Abcam 32,127, RRID AB 2286949, 1/2000) were incubated overnight at 4°C followed by 1 h of incubation with a secondary antibody (Abcam 150,077, RRID AB2630356 or RRID AB 2650601 150,116, 1/1000) and DAPI (0.5 μg/mL) at RT.

For counting of RGCs, Brn3-a immuno-stained sections were scanned using a NanoZoomer 2.0 HT (Hamamatsu). Automatic counting was performed using the ORBIT Image Analysis software package. Three to four retinal sections were analyzed for each mouse of each genotype.

GFAP, Iba1, and synaptophysin immuno-stained retinal sections were imaged using a Leica SP8 confocal microscope. GFAP staining (one section per mouse) was examined on z-stacked images (~30 planes/10 μm-thick retinal section). Iba1 staining was investigated on three-dimensional images using the Imaris XT software package (Bitplane AG). Iba1-positive cells were counted on six retinal sections per mouse. The total process length of 18–20 Iba1-positive cells per mouse was measured using the Imaris Filament Tracer module. For synaptophysin experiments, all the confocal microscope settings were kept the same across all compared samples. Labeling was quantified on z-stacked images using ImageJ. The analysis was performed for each section (one per mouse) on a defined ROI corresponding to the entire inner or outer plexiform layer. For all images, the same threshold was applied, and the mean gray value was measured for each ROI and normalized to the area of the corresponding ROI.

### 2.4. Transmission electron microscopy

Optic nerves were isolated from freshly euthanized mice (Pozyuchenko et al., 2020), then fixed with 2.5% glutaraldehyde and 2% paraformaldehyde in cacodylate buffer (0.1 M, pH 7.2, EMS, Hatfield, PA) for 2 h and post-fixed at 4°C with 1% osmium tetraoxide in the same buffer. Samples were treated for 1 h with 1% aqueous uranyl acetate, dehydrated in a graded ethanol series, and embedded in EMBed-812 resin (EMS). After 48 h of polymerization at 60°C, ultrathin sections (80 nm) were made and these were mounted on 200-mesh formvar–carbon-coated copper grids, stained with Uranyless (Delta Microscopies) and 3% Reynolds lead citrate (Chromalys). Grids were examined using a TEM (Jeol JEM-1400, JEOL Inc) at 80 kV. Images (5 areas within one optic nerve section per mouse) were acquired using a digital camera (Gatan Orius, Gatan Inc., Pleasanton, CA, United States) at 2000× magnification. Axonal phenotypes were considered to be abnormal if they presented with clumping of neurofilament, dense axoplasm, empty axoplasm, or myelin abnormalities (see Supplementary Figure S1). The number of axons and their area were analyzed using the ImageJ software package. Myelin thickness (one per axon) was measured by manually drawing a perpendicular line across a compact area of myelin sheath, which was measured using the Analyze section of ImageJ. The inner (axon) to outer (axon plus myelin) diameters of the myelinated axons were obtained by measuring the Feret’s diameter (ImageJ), and these measurements were used to calculate the myelin g-ratio by dividing the inner diameter by the outer diameter.

### 2.5. Statistical analysis

Results are expressed in the form mean ± SEM. Statistical analyses were performed using the GraphPad Prism software package. For comparisons between OPA1-mutated and control mice, we performed either unpaired Student’s *t*-tests or Mann–Whitney tests, depending on the normality of the distribution of the data, which was checked using the Kolmogorov–Smirnov normality test. The type of statistical test used in each case, as well as the corresponding *p*-values, is found in the relevant figure legend.

## 3. Results

### 3.1. Reduced OPA1 protein levels in the OPA1^329-355del^ C57BL/6J mouse model

**Figure 1:**
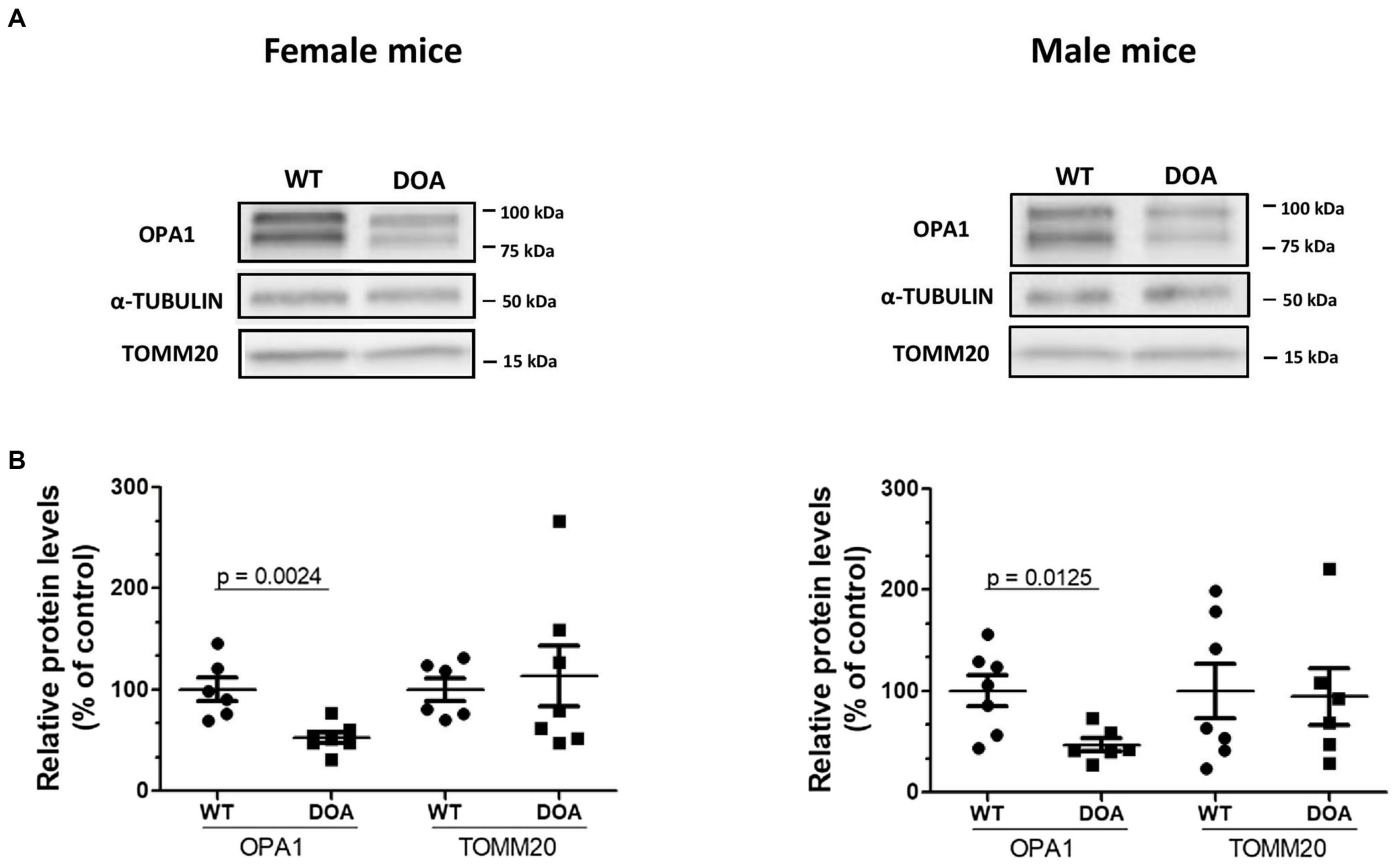
Reduced OPA1 protein levels in the OPA1^329-355del^ C57BL/6 J mouse model. **(A)** Representative immunoblots showing OPA1, α-TUBULIN, and TOMM20 protein levels in the retinas of 11-month-old WT and DOA mice (females, left panel; males, right panel). **(B)** Protein levels of the mitochondrial proteins OPA1 and TOMM20 relative to α-TUBULIN in the retinas of female (left panel) and male (right panel) DOA mice and their littermate controls. Quantification was performed using Image Lab (Biorad). Each raw data point for WT and DOA is normalized to the mean of the WT values. The results plotted are mean percentages of control values ± SEM. Statistical analysis (*n* = 6–7 mice/group) was performed on raw data using unpaired *t*-tests to compare WT and DOA mice on OPA1 and TOMM20 levels. *p* values are indicated on the graph when significant (<0.05). DOA: OPA1^329-355del^ mice with C57BL/6 J pure genetic background. WT: littermate controls.

OPA1 protein levels in the retinas of 11-month-old male and female OPA1^329-355del^ C57BL/6 J (DOA) mice and their wild-type (WT) littermates were estimated by Western blot (Figure 1). In all cases, two main bands were detected at 100 kDa and 75 kDa, corresponding to the long and short isoforms of the protein, respectively (Figure 1A). However, the total OPA1 protein level was reduced to 52% ± 5.3 (Figure 1B, left) and 47% ± 6.6 (Figure 1B, right) in DOA females and males, respectively, relative to their WT littermates. OPA1 reduction was not a consequence of decreased mitochondrial biomass, since mitochondrial TOMM20 protein levels were equivalent in DOA and WT mice for both sexes (Figure 1B). Hence, we showed that the c.1065 + 5G → A *Opa1* mutation leads to OPA1 haploinsufficiency, i.e., ~50% reduction in OPA1 protein levels, in both female and male DOA mice of the pure C57BL/6 J genetic background.

### 3.2. No loss of retinal ganglion cells in the OPA1^329-355del^ C57BL/6J mouse model

**Figure 2:**
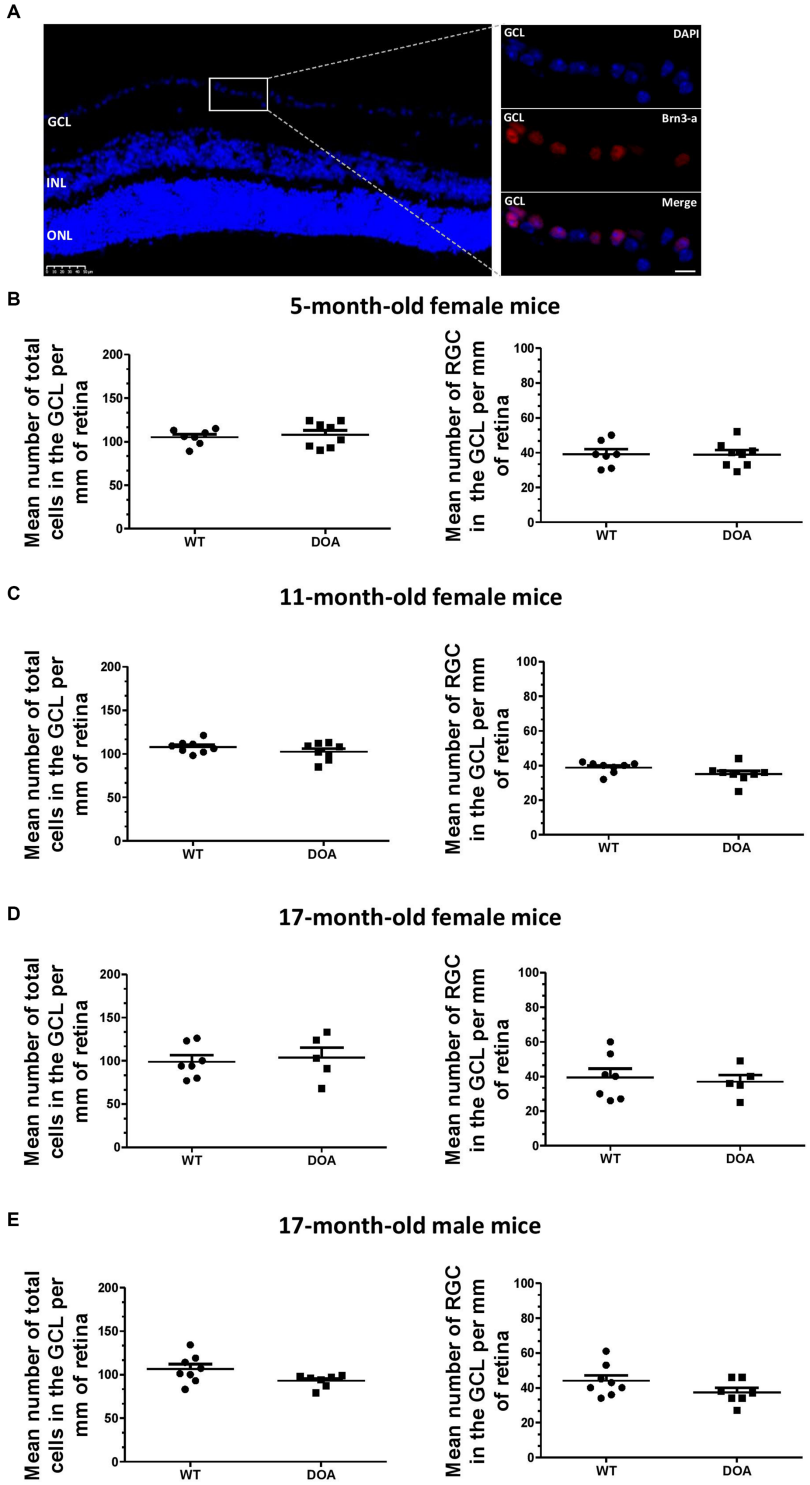
No retinal ganglion cell loss in the OPA1^329-355del^ C57BL/6 J mouse model. **(A)** Mouse retinal sections were immunolabeled with an anti-Brn3-a antibody and counterstained with DAPI. A representative image obtained for a 17-month-old WT mouse is shown. Left panel: DAPI staining showing the different nuclear layers (GCL, ganglion cell layer; INL, inner nuclear layer; ONL, outer nuclear layer). The scale bar represents 50 μm. The white rectangle indicates the magnified region of the GCL in the right panel. The upper right panel corresponds to DAPI staining (blue), the middle right panel to Brn3-a staining for labeling of RGCs (red, Alexa Fluor 594 goat anti-mouse IgG), and the lower right panel to merged images. The scale bar represents 20 μm. **(B–E)** Quantification of total number of cells (DAPI staining, left) and number of retinal ganglion cells (RGC) (Brn3-a staining, right) in the GCL of 5-, 11-, and 17-month-old female mice [**(B–D)**, respectively] and 17-month-old males **(E)**. Results were normalized by GCL length and values plotted are means ± SEM. Statistical analyses (*n* = 5–8 mice/group) were performed using the Mann–Whitney test **(B,D,E)** and an unpaired *t*-test **(C)** to compare WT and DOA mice on total cells in the GCL or number of RGCs, for each age and sex group. In all cases, the *p* value was >0.05. DOA: OPA1^329-355del^ mice with C57BL/6 J pure genetic background. WT: littermate controls.

Total cell loss and specific retinal ganglion cell (RGC) loss in the ganglion cell layer (GCL) were evaluated by DAPI staining and immunodetection using antibodies against Brn3-a (a specific marker of RGCs) on retinal sections, respectively (Figure 2A). There was no difference either in the total number of cells (Figures 2B–D, left) or in the number of RGCs (Figures 2B–D, right) in 5-, 11-, or 17- month-old female DOA mice, or in 17-month-old male DOA mice (Figure 2E), when compared to their littermate controls (WT). Thus, the c.1065 + 5G → A *Opa1* mutation did not induce a reduction in the number of RGCs even at an advanced stage of the disease, in the context of the pure C57BL/6 J genetic background.

### 3.3. No retinal inflammation in the OPA1^329-355del^ C57BL/6J mouse model

**Figure 3:**
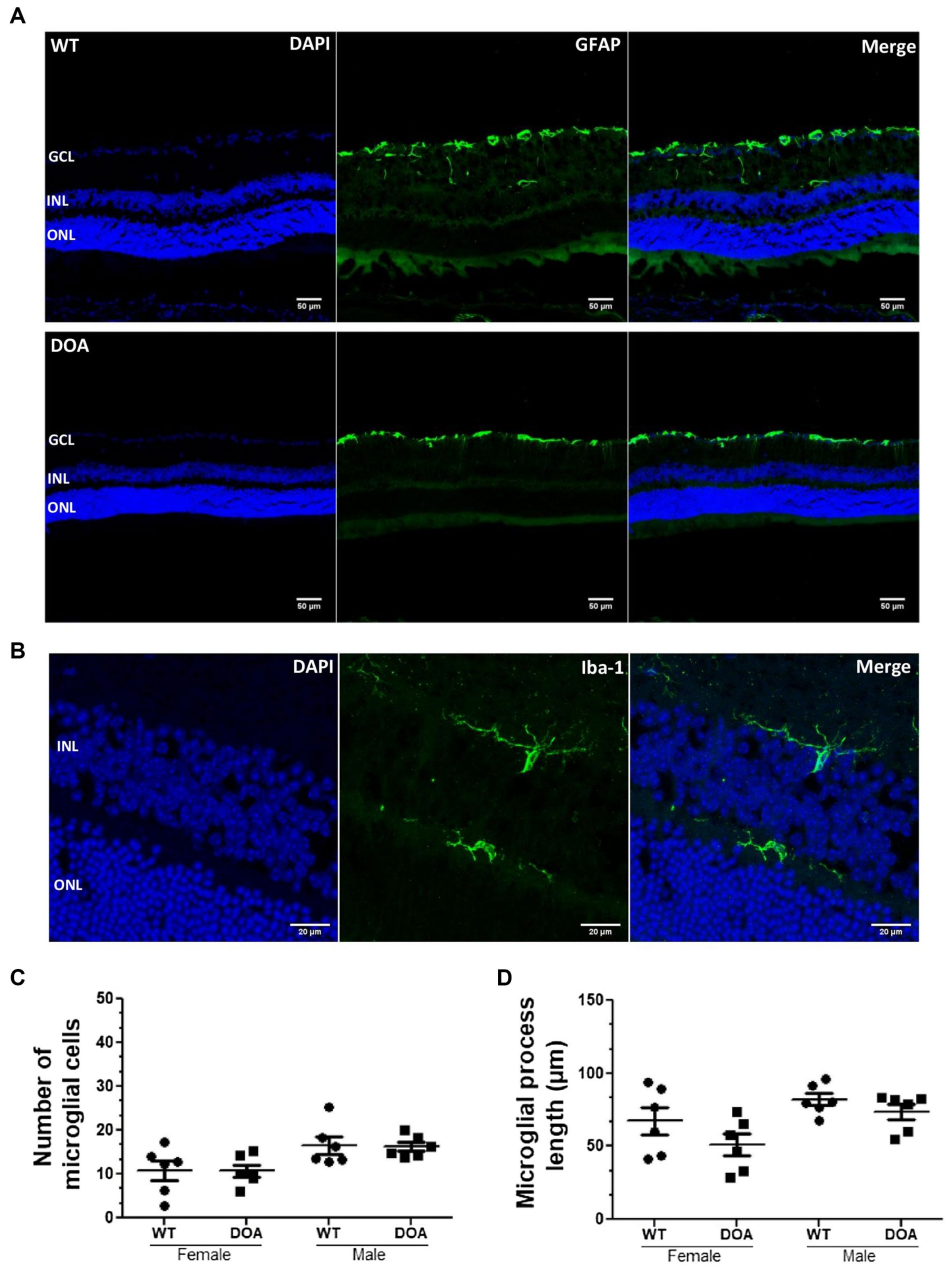
No retinal inflammation in the OPA1^329-355del^ C57BL/6 J mouse model. **(A)** Mouse retinal sections were immunolabeled with an anti-GFAP antibody (green, Alexa Fluor 488 goat anti-rabbit IgG) and counterstained with DAPI (blue). Representative images obtained for 11-month-old WT (top) and DOA (bottom) mice are shown. Scale bar, 50 μm. GCL, ganglion cell layer; INL, inner nuclear layer; ONL, outer nuclear layer. **(B)** Retinal sections were immunolabeled using an antibody against Iba-1 (green, Alexa Fluor 488 goat anti-rabbit IgG) and counterstained with DAPI (blue). The staining was performed on retinal sections of 17-month-old female and male WT and DOA mice. Representative staining for DOA female mice is shown. Scale bar, 20 μm. **(C)** Number of Iba-1-positive microglial cells throughout retinal sections of 17-month-old female and male DOA and WT mice. The values plotted are means ± SEM. Statistical analyses (*n* = 6 mice/group) were performed using the Mann–Whitney test to compare WT and DOA mice on the number of microglial cells, either in females or in males. In all cases, the *p* value was >0.05. **(D)** Length of microglial processes in the retinal sections of 17-month-old male and female DOA and WT mice. The values plotted are means ± SEM. Statistical analyses (*n* = 6 mice/group) were performed using the Mann–Whitney test to compare WT and DOA mice on the length of microglial processes, either in females or in males. In all cases, the *p* value was >0.05. DOA: OPA1^329-355del^ mice with C57BL/6 J pure genetic background. WT: littermate controls.

Inflammatory status in the retina was assessed by immunostaining of astrocytes and Müller cells (GFAP staining) as well as microglial cells (Iba1 staining) (Figures 3A,B). GFAP-positive cells were found in the inner part of the retina (the GCL and retinal nerve fiber layer), with no morphological or distribution changes in either sex or at any of the different ages examined in DOA mice when compared to their littermate WT controls (Figure 3A; other data not shown). Identical numbers of microglial cells (Figure 3C) and similar total process lengths (Figure 3D) were observed in the retinas of 17-month-old DOA and WT mice. Taken together, these results suggest no inflammation at any age for either sex in the OPA1^329-355del^ C57BL/6 J mouse model.

### 3.4. Decreased synaptophysin expression in the IPL of the OPA1^329-355del^ C57BL/6J mouse model

**Figure 4:**
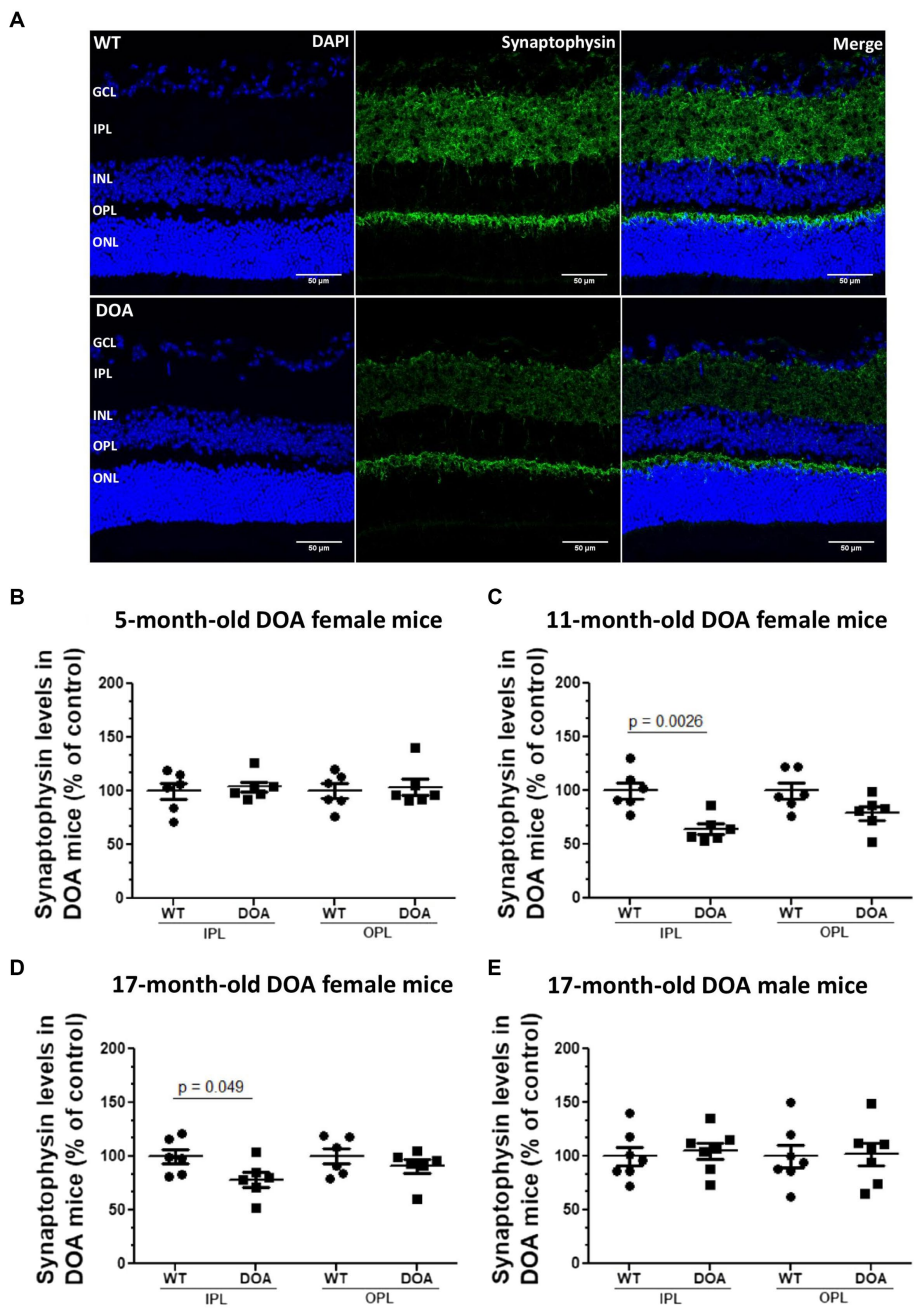
Decreased synaptophysin expression in the IPL in the OPA1^329-355del^ C57BL/6 J mouse model. **(A)** Retinal sections of 5-, 11-, and 17-month-old female and 17-month-old male mice were immunolabeled using an antibody against synaptophysin (green, Alexa Fluor 488 goat anti-rabbit IgG) and counterstained with DAPI (blue). Representative images of 11-month-old female WT (top) and DOA (bottom) mice are shown. Scale bar, 50 μm. GCL, ganglion cell layer; IPL, inner plexiform layer; INL, inner nuclear layer; OPL, outer plexiform layer; ONL, outer nuclear layer. **(B–E)** Synaptophysin expression levels within the inner and outer plexiform layer for **(B)** 5-month-old, **(C)** 11-month old, **(D)** 17-month-old female mice, and **(E)** 17-month-old male mice. Each raw data point for WT and DOA was normalized to the average WT value. The results plotted are mean percentages of control values ± SEM. Statistical analyses (*n* = 6–7 mice/group) was performed on raw data using unpaired *t*-tests to compare WT and DOA mice on synaptophysin levels for each age and sex group. *p* values are indicated on the graph when significant (<0.05). DOA: OPA1^329-355del^ mice with C57BL/6 J pure genetic background. WT: littermate controls.

To evaluate synaptic integrity, we performed synaptophysin immunostaining, as an index of presynaptic compartments, on retinal sections (Figure 4). As expected, synaptophysin immunoreactivity was found in the inner and outer plexiform layers (IPL and OPL) (Figure 4A). For the OPL, no difference was observed regardless of the age, sex, or genotype of the mice. In the IPL, a significant decrease was observed in 11- and 17-month-old female DOA mice, reaching 64% ± 4.9 (Figure 4C) and 78% ± 7 (Figure 4D) relative to the control, respectively. This decrease was not found in male DOA mice. These results show for the first time a sex-dependent alteration of the presynaptic compartment in the IPL of OPA1^329-355del^ C57BL/6 J mice.

### 3.5. No abnormalities in the optic nerve of the OPA1^329-355del^ C57BL/6J mouse model

**Figure 5:**
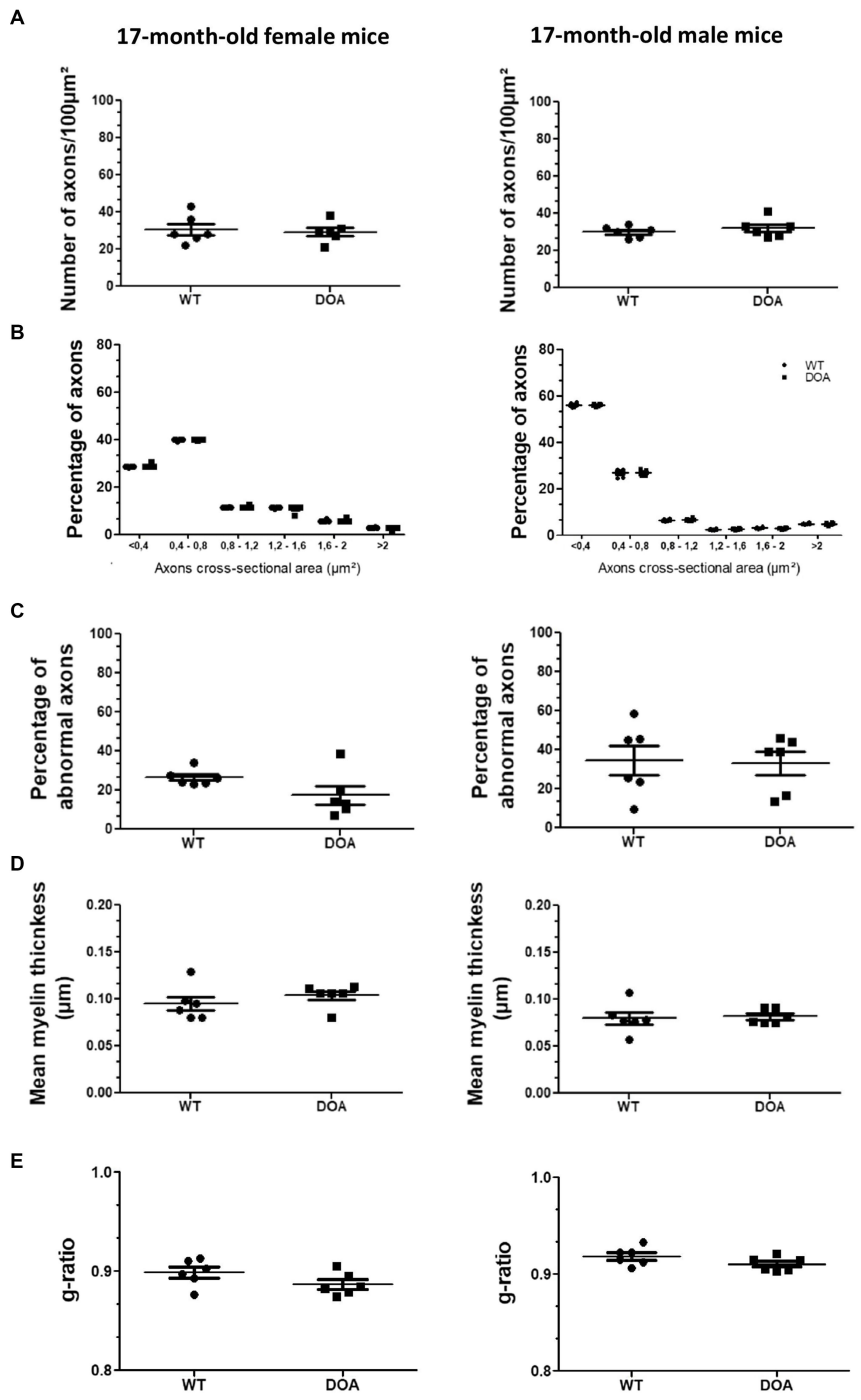
No abnormalities in the optic nerve of OPA1^329-355del^ C57BL/6 J mice. **(A)** Number of axons in the optic nerves of 17-month-old female (left) and male (right) WT and DOA mice. The results plotted are mean number per 100μm^2^ ± SEM. Statistical analyses (*n* = 6 mice/group) were performed using the Mann–Whitney test to compare WT and DOA mice on the number of axons, either in females or in males. In each case, the *p* value was >0.05. **(B)** Classification of optic nerve axons according to their cross-sectional area in 17-month-old female (left) and male (right) WT and DOA mice. Results plotted are mean percentages of total axon numbers ± SEM. Statistical analyses (*n* = 6 mice/group) were performed using the Mann–Whitney test to compare WT and DOA mice on the area of axons, either in females or in males. In each case, the *p* value was >0.05. **(C)** Percentages of axons presenting an abnormal phenotype in the optic nerves of 17-month-old female (left) and male (right) WT and DOA mice. The results plotted are mean percentages of total axons ± SEM. Statistical analyses (*n* = 6 mice/group) were performed using the Mann–Whitney test to compare WT and DOA mice on the rate of occurrence of abnormal axon phenotypes, either in females or in males. In each case, the *p* value was >0.05. **(D)** Mean myelin thickness of axons in the optic nerves of 17-month-old female (left) and male (right) WT and DOA mice. The results plotted are mean percentages ± SEM. Statistical analyses (*n* = 6 mice/group) were performed using the Mann–Whitney test to compare WT and DOA mice on myelin thickness, either in females or in males. In each case, the *p* value was >0.05. **(E)** Mean myelin g-ratio in the optic nerves of 17-month-old female (left) and male (right) WT and DOA mice. The results plotted are mean percentages ± SEM. Statistical analyses (*n* = 6 mice/group) were performed using the Mann–Whitney test to compare WT and DOA mice on g-ratio, either in females or in males. In each case, the *p* value was >0.05. DOA: OPA1^329-355del^ mice with C57BL/6 J pure genetic background. WT: littermate controls.

An evaluation of optic nerve integrity was conducted via transmission electron microscopy (Figure 5). We first quantified the number of axons (Figure 5A) and assessed their diameter (Figure 5B). As exemplified at 17-months of age, none of these parameters turned out to differ between DOA and WT mice, regardless of age or sex (females: left panels; males: right panels). We then carried out a morphological study of the axons, looking for abnormal phenotypes, including neurofilament aggregation, dense axoplasm, empty axons, and aberrant myelination (Supplementary Figure S1). Abnormal axonal phenotypes were observed at the same frequency in both DOA and WT mice, regardless of age or sex (Figure 5C). Analyses of the myelin sheath showed no change in either myelin thickness (Figure 5D) or the myelin g-ratio (Figure 5E) in DOA mice, at any age in either sex and regardless of genotype, as compared to WT mice. Therefore, no reduction in the number of RGC axons or increase in the incidence of abnormal axon phenotype was observed in OPA1^329-355del^ C57BL/6 J mice.

## 4. Discussion

Optic atrophy is principally caused by mutations of genes encoding mitochondrial proteins or by environmental conditions affecting the mitochondria. The two main forms of hereditary optic atrophy are Leber hereditary optic neuropathy (LHON), which is primarily caused by mutations in three mtDNA-encoded subunits of the mitochondrial respiratory complex I, and dominant optic atrophy (DOA), mainly attributable to mutations of the nuclear gene *OPA1*. These two pathologies present with incomplete penetrance and highly variable expressivity, suggesting that other genetic and/or environmental factors might influence the onset and the severity of the disease (Caporali et al., 2017). For LHON, several susceptibility factors have been identified, including excessive tobacco and alcohol consumption and exposure to specific toxins and drugs, but also mtDNA haplogroup or genetic variation in the nuclear genome (Hudson et al., 2007; Kirkman et al., 2009; Yu et al., 2020). Thus far, these complex genetics–environment interactions remain poorly understood in the case of DOA, and only a small number of intragenic OPA1 modifiers have been shown to modulate disease manifestations, while it is unclear whether mtDNA background is involved (Han et al., 2006; Pierron et al., 2009; Bonifert et al., 2014).

To evaluate the influence of genetic background on DOA expressivity, we switched the DOA mouse model (Opa1^329-355del^) previously described by Alavi and collaborators (Alavi et al., 2007) from a mixed (C3H; C57BL/6 J) genetic background to a pure C57BL/6 J background. This model features a splice site mutation in the *Opa1* gene (c.1065 + 5G → A), resulting in OPA1 haploinsufficiency. In our study, the presence of the *Opa1* mutation and the 50% reduction in protein levels were systematically confirmed by PCR and Western blot, respectively.

While OPA1 haploinsufficiency occurs in mice of both genetic backgrounds, the consequences for retinal and optic nerve integrity are dramatically different. Indeed, in the mixed genetic background Opa1^329-355del^ model, degeneration of RGCs has been found to occur progressively (15, 25, and 50% at 2, 9, and 13 months of age, respectively) (Alavi et al., 2007; Heiduschka et al., 2010; Nguyen et al., 2011). In contrast, in the pure context, OPA1 deficiency did not lead to RGC loss, regardless of the age or sex of the mice. In case of the mixed genetic background, the number of RGCs was estimated after injection of retrograde tracer into the superior colliculi (Alavi et al., 2007; Heiduschka et al., 2010; Nguyen et al., 2011), while we labeled RGCs with an antibody directed against Brn3-a. This antibody has previously been shown to label the nuclei of the vast majority of tracer retro-labeled RGCs (~ 92%) and to detect more than 85% of RGCs in mice (Galindo-Romero et al., 2011). However, we cannot rule out a possible specific loss of a minority of Brn3-a negative RGCs, representing less than 6% of the cellular population of the GCL (Jeon et al., 1998). It should be noted that the intrinsically photosensitive RGCs (ip-RGCs), which express melanopsin and account for a portion of Brn3-a-negative RGCs, are spared both in DOA patients (la Morgia et al., 2010) and in the mixed mouse model of DOA (González-Menéndez et al., 2015). RGC loss has been found to be accompanied by both astroglial and microglial activation in the case of the mixed genetic background (Nguyen et al., 2011), but evidence for this was not found in mice of the pure genetic background. The phenotypic outcomes of the optic nerve were also different. We did not observe evidence for any change in number, size, or integrity of axons, or in the amount of myelin, in the case of the pure C57BL/6 J background, while in mixed background mice, axonal demyelination and degeneration (swelling and distorted shapes) have been described from 8 months of age (Alavi et al., 2007), triggering 47% axonal loss at 18 months of age; this has been found to be even more pronounced for small-caliber axons (González-Menéndez et al., 2015). Interestingly, similar discrepancies have been observed in other DOA mouse models also based on *OPA1* gene mutations leading to OPA1 haploinsufficiency. In particular, RGC loss does occur upon OPA1 haploinsufficiency in the Opa1^delTTAG^ model (Sarzi et al., 2012, 2016), but is not observed in the Opa1^Q285STOP^ model (Davies et al., 2007). Likewise, severe axonal damage has been observed in Opa1^delTTAG^ mice, while Opa1^Q285STOP^ mice display only a late and mild axonal damage phenotype.

Although different mutations in the *Opa1* gene are present in each of the three DOA mouse models, all trigger OPA1 haploinsufficiency, suggesting similar pathological consequences. However, all these models were developed in the context of different genetic backgrounds: mixed C3H; C57BL/6 J in the case of the Opa1^329-355del^ model, mixed 129/SvPas; C57BL/6 J for the Opa1^delTTAG^ model, and C57BL/6 after outcrossing over at least 4 to 5 generations for the Opa1^Q285STOP^ model. Hence, we assume that the occurrence or absence of retinal and optic nerve defects depends on the presence of aggravating or protective genetic co-factors. This hypothesis is largely supported by the results we obtained by changing the genetic background for the same mutation. Overall, the observed differences suggest the contribution of secondary modifying factors. Comparison of the C57BL/6 J reference genome with 36 other sequenced mouse strains indicates numerous coding genes whose loss-of-function mutations may contribute to phenotypic differences (Timmermans et al., 2017). Among these, C57BL/6 J mice display a spontaneous mutation in the *Nnt* gene, encoding nicotinamide nucleotide transhydrogenase, which alters the redox state in hepatic mitochondria, inducing catalase upregulation for maintenance of physiological H_2_O_2_ levels (Toye et al., 2005; Dogar et al., 2020). Such an adaptation, if occurring in the retina, could provide protection against the redox imbalance that arises in OPA1 haploinsufficiency (Millet et al., 2016).

In the Opa1^Q285STOP^ mouse model, no evidence has been found for the loss of RGCs; instead, altered synaptic connectivity and dendritic arborization are observed (Williams et al., 2010, 2012). In our study, we highlighted a significant decrease in the levels of the presynaptic protein synaptophysin, specifically in the IPL of DOA mice, where RGC dendrites form connections with bipolar and amacrine cells. Overall, these data suggest that OPA1 deficiency may first impair RGC function through early synaptic and dendritic alterations, ultimately leading to neuronal degeneration and cell death upon exposure to additional stresses that could be induced by genetic or environmental factors. Accordingly, we have previously shown that OPA1 haploinsufficiency in primary neuronal culture is associated with decreased synaptic protein content and number of synapses, but does not induce neuronal death *per se*, whereas additional exogenous stresses increase susceptibility to cell death (Bertholet et al., 2013; Lassus et al., 2016; Millet et al., 2016).

While most previous studies agree that both sexes are similarly affected in DOA (or do not consider sex), we report a sex-dependent synaptic phenotype. This is consistent with data recently obtained for the Opa1^Q285STOP^ model, showing that female mice are more prone to impaired dendritic arborization and spine density in the hippocampal neurons, ultimately leading to synaptic disruption (Bevan et al., 2020). Moreover, in the Opa1^delTTAG^ mouse model, RGC loss and reduced visual acuity have been observed earlier in female mice (5 months) than in male mice (11 months) (Sarzi et al., 2016). Interestingly, in humans, women patients also suffer from early disease onset, and maternal inheritance of OPA1 mutations increases susceptibility to the development of a syndromic form of DOA (Sarzi et al., 2016; Ham et al., 2019). Sex differences have already been demonstrated in ocular diseases such as LHON, and also in other neurodegenerative diseases (Vegeto et al., 2020; Korpole et al., 2022). It would therefore be relevant to consider sex as a susceptibility factor that may contribute to the pathogenesis of DOA.

To conclude, our work showed for the first time that genetic background influences DOA expressivity in mice, since the same *Opa1* mutation was found to lead to distinct phenotypes in different genetic environments. This parallels the heterogeneity observed in patients, given that some mutations have consequences only in some individuals (incomplete penetrance) or present a range of forms of symptomatic expression between individuals (variable expressivity). Recently, several genes whose depletion can counteract the impairments to mitochondrial dynamics induced by OPA1 deficiency have been identified and could thus be tested as genetic modifying factors (Cretin et al., 2021). In this context, we provide a new mouse model that, given its better fit to asymptomatic patients, offer the possibility of screening for environmental and genetic modulators that could worsen the disease. Finally, we also demonstrate a sex-dependent disruption in RGC connectivity, opening up a pathway to new clinical considerations for diagnosis as well as a therapeutic window for action before the irreversible phase of the disease.

## Data availability statement

The original contributions presented in the study are included in the article/Supplementary material, further inquiries can be directed to the corresponding authors.

## Ethics statement

Ethical approval was not required for the study involving animals in accordance with the local legislation and institutional requirements because under European regulations, only the use of animals in experimental procedures requires the approval of an ethics committee. Article R214-89 of the French Rural Code specifies that “The killing of animals, for the sole purpose of using their organs or tissues, in accordance with a method defined by joint order of the Minister for Agriculture and the Minister for Research, is not considered to be an experimental procedure.” This is the case for the present project, which has therefore not been evaluated by the ethics committee. However, the animals were produced and bred in an approved animal facility (approval number 31-555-011) by staff with all the necessary qualifications.

## Author contributions

DA, VG, PP, FK, MA, and MS performed the experiments. DA and PB designed the experiments. BW provided the original DOA mice. MB was in charge of the new DOA mouse model. PB supervised the work. DA, MS, and PB wrote the article. All authors contributed to the article and approved the submitted version.

## Funding

This work was supported by CNRS, Université Toulouse III, Aviesan Neurosciences/Unadev (17UU054-00, 20UU186), Retina France (192127, 177366), and AFM (20277). DA was the recipient of a PhD fellowship from Fondation de France (00099527) and MA was the recipient of a Postdoctoral Fellowship-Becas Chile CONICYT and ANID Fondecyt Iniciación (11220120).

## Conflict of interest

The authors declare that the research was conducted in the absence of any commercial or financial relationships that could be construed as a potential conflict of interest.

## Publisher’s note

All claims expressed in this article are solely those of the authors and do not necessarily represent those of their affiliated organizations, or those of the publisher, the editors and the reviewers. Any product that may be evaluated in this article, or claim that may be made by its manufacturer, is not guaranteed or endorsed by the publisher.
