# Supplementary material for: Genetic background modulates phenotypic expressivity in OPA1 mutated mice, relevance to DOA pathogenesis
Source: Front Mol Neurosci. 2023 Sep 6;16:1241222. doi: 10.3389/fnmol.2023.1241222 (PMC10510408; doi:10.3389/fnmol.2023.1241222)
Supplement: Supplementary file 1 [file Data_Sheet_1.docx]

### **Supplementary Figure 1 legend. Axonal phenotypes in mouse optic nerves.**

Representative phenotypes of axons observed by electron microscopy within the optic nerves of WT and DOA mice. Abnormal axonal phenotypes include neurofilament aggregation (white asterisks), dense axoplasm, empty axons, and aberrant myelination, either focal demyelination (white arrowheads) or excess of myelin (white arrows).
