# Supplementary figures and images for: Genetic background modulates phenotypic expressivity in OPA1 mutated mice, relevance to DOA pathogenesis
Source: Front Mol Neurosci. 2023 Sep 6;16:1241222. doi: 10.3389/fnmol.2023.1241222 (PMC10510408; doi:10.3389/fnmol.2023.1241222)

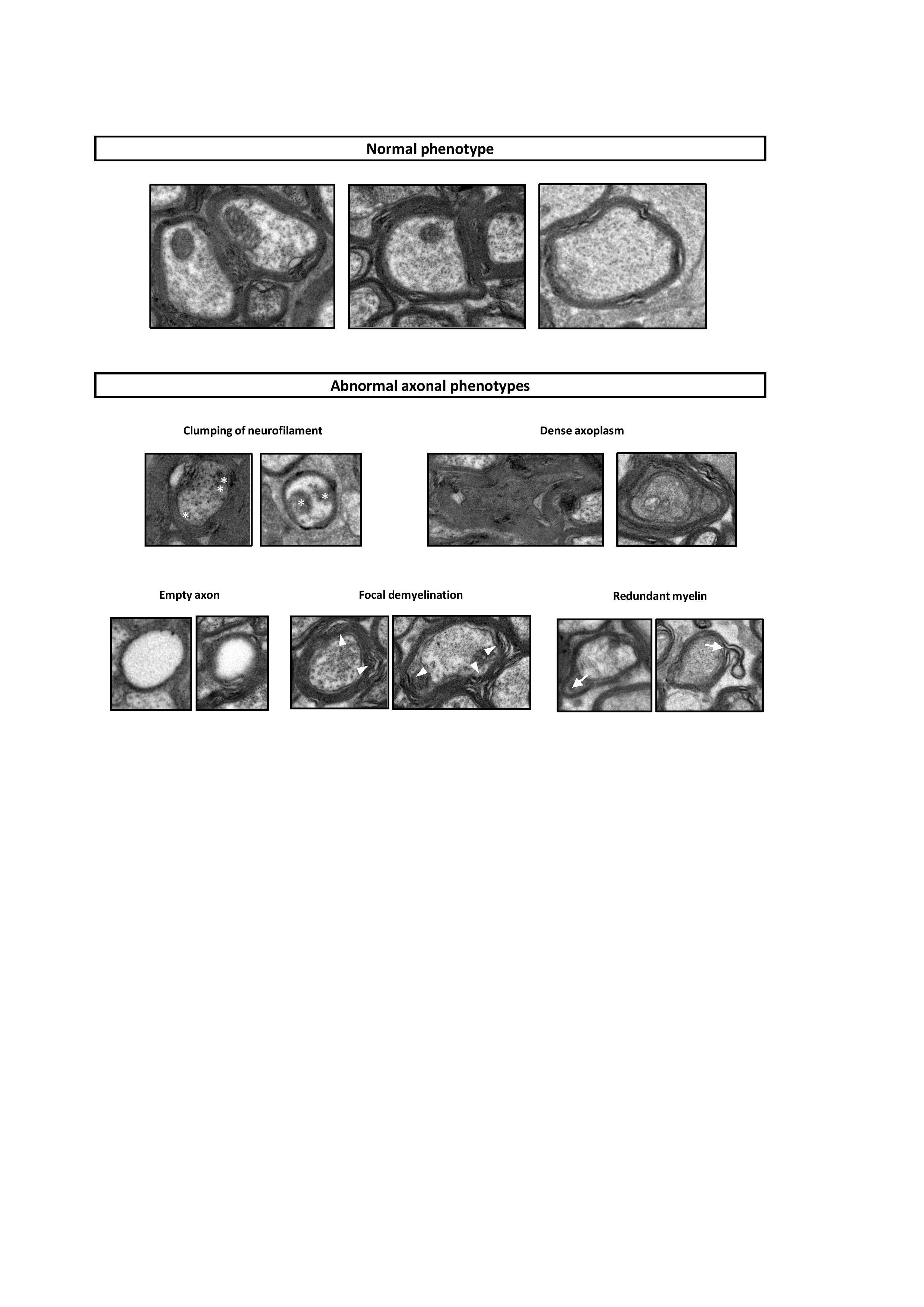

Supplement: Supplementary file 2 [file Image_1.jpg]
